# Editorial: Application of emerging technologies in the diagnosis and treatment of patients with brain tumors: new frontiers in imaging for neuro-oncology

**DOI:** 10.3389/fonc.2025.1625256

**Published:** 2025-06-03

**Authors:** Domenico Aquino, Elena De Martin, Camilla Russo

**Affiliations:** ^1^ Neuroradiology, Fondazione IRCCS Istituto Neurologico Carlo Besta, Milan, Italy; ^2^ Health Department, Fondazione IRCCS Istituto Neurologico Carlo Besta, Milan, Italy; ^3^ Department of Pediatric Neurosciences, Neuroradiology, Santobono-Pausilipon Children’s Hospital, Naples, Italy

**Keywords:** MRI, brain, tumor, radiomic, prognostic

Accurate prognostication across brain tumor types — particularly those with the poorest outcomes, such as high-grade gliomas and especially glioblastoma multiforme (GBM) — remains a central challenge in neuro-oncology. Although advances in surgical techniques, radiotherapy, and systemic therapies have extended survival for some patients, outcomes still vary widely and are difficult to predict based on clinical and molecular data alone. Conventional prognostic models typically incorporate factors such as age, performance status, tumor location, histopathological grade, and molecular alterations (e.g., MGMT promoter methylation, IDH mutation). However, these measures often fail to capture the full extent of biological complexity and spatial heterogeneity within tumors that underlie treatment resistance and progression. Consequently, there is a pressing need for reliable, noninvasive biomarkers that more comprehensively reflect tumor biology, facilitate risk stratification, guide individualized treatment planning, and ultimately improve patient outcomes.

In this context, quantitative neuro-imaging has emerged as a valuable adjunct to traditional assessment, with research concentrating principally on brain tumors with a worse prognosis. Multiparametric MRI — including contrast-enhanced T1-weighted, T2-weighted, FLAIR, diffusion-weighted, and perfusion sequences — provides complementary information on tumor morphology, cellularity, and vascular characteristics. Radiomic analysis further enhances this approach by extracting high-dimensional quantitative features (intensity, shape, texture, and wavelet-based metrics) from defined regions of interest, transforming images into data-rich profiles and underscoring the potential of imaging-derived biomarkers to improve prognostic accuracy and tailor therapy across diverse brain tumor entities. Notably, Kickingereder et al. used radiomic analysis to identify imaging phenotypes in high grade gliomas associated with patient prognosis; by extracting high-dimensional imaging features, they demonstrated that certain radiomic profiles correlate with overall survival, independent of established clinical variables, with a prognostic accuracy superior when compared with clinical and conventional imaging models (1). Similarly, the integration of quantitative perfusion MRI parameters with genetic profiling and molecular insights has proven to enhance tumor characterization, more accurate prognostication, and allow for more tailored therapeutic strategies. Such synergy paves the way for a more refined and individualized model of care in neuro-oncology, moving beyond traditional one-size-fits-all paradigm and toward a future of truly personalized medicine (2). Thus, there are several studies that illustrate how combining radiomic features with molecular and clinical data yields composite models that capture complementary dimensions of tumor biology, offering refined prognostic insights and potential predictive value for targeted therapies. Clinical applications of diffusion-MRI, which include among others diffusion-weighted imaging (DWI) with apparent diffusion coefficient (ADC) maps, diffusion tensor imaging (DTI) and diffusion kurtosis imaging (DKI), have further illuminated the prognostic significance of cellular density and extracellular matrix composition in gliomas. For instance, Li et al. investigated the prognostic value of DKI in GBM, demonstrating how higher mean kurtosis values (reflecting microstructural tissue complexity) were significantly associated with longer overall survival (3). In parallel, Chen et al. assessed how ADC histogram analysis is able to provide innovative insights into the MGMT and TERT molecular characterization in patients newly diagnosed with GBM, enhancing prognostic stratification (4). These findings support the notion that ADC-derived imaging biomarkers reflect underlying histopathological and molecular traits, enabling noninvasive *in vivo* phenotyping of tumor aggressiveness. Advanced perfusion imaging metrics also have prognostic and predictive implications. Dynamic susceptibility contrast MRI–derived cerebral blood volume maps correlate with microvascular density and angiogenic activity. In the context of antiangiogenic therapy, Kickingereder et al. identified pre-treatment radiomic features that predicted progression-free and overall survival in recurrent GBM patients treated with bevacizumab (5). The integration of perfusion radiomics and treatment response modeling highlights how imaging biomarkers can guide therapeutic decision-making, identifying patients most likely to benefit from targeted agents. Machine learning and deep learning techniques have accelerated the discovery of prognostic imaging signatures by automating tumor segmentation, feature extraction, and pattern recognition. Deep learning has significantly transformed the automated segmentation and classification of intracranial tumors, showing particularly promising performance in the context of gliomas, and offering notable advantages in terms of time efficiency and resource optimization; in particular, deep-learning–based segmentation algorithms improve the reproducibility of region of interest delineation, a critical factor for multicenter studies (6). Lao et al. employed transfer learning to derive deep radiomic features from pre-trained convolutional neural networks, constructing a nomogram that outperformed traditional clinical risk factors and handcrafted radiomic features for survival prediction (7). These deep features capture complex, hierarchical image representations that may elude conventional radiomic pipelines. Radiogenomic approaches integrate imaging phenotypes with genomic and transcriptomic landscapes, providing mechanistic insights and enhancing prognostic models. Radiomics has been applied to various areas of neuro-oncology, with particular success in the differential diagnosis and classification of brain tumors (8). Kickingereder et al. used radiomic subtyping to classify GBM tumors into phenotypic clusters associated with differential survival, revealing that specific subtypes—characterized by heterogeneous texture and angiogenic features—portend poorer outcomes (9). A study by Qi et al. demonstrated that specific imaging features correlated with molecular subtypes of GBM, such as proneural and mesenchymal profiles; this spatial distinction, combined with differences in imaging characteristics, allows for the noninvasive prediction of molecular subtypes using MRI data (10). Integrative models that combine radiomics, genomics, and clinical variables have shown superior prognostic performance compared with single-modality models, underscoring the value of multimodal data fusion in precision neuro-oncology. Despite these advances, several challenges impede the clinical translation of prognostic neuro-imaging biomarkers. Variability in MRI acquisition protocols, scanner manufacturers, and imaging parameters introduces heterogeneity that can compromise feature stability. Initiatives to standardize imaging protocols and develop digital reference objects for quality assurance are critical to ensure reproducibility. Segmentation remains labor-intensive and subject to interobserver variability; robust, validated automated or semi-automated segmentation tools are needed to enable widespread adoption. Retrospective study designs and small sample sizes limit generalizability; large-scale, multicenter, prospective studies with standardized imaging and outcome metrics are required for validation. Finally, regulatory pathways for imaging biomarkers demand clear demonstration of clinical utility and cost-effectiveness. In summary, quantitative neuro-imaging and radiomics have reshaped the landscape of prognostication in brain tumors, offering noninvasive insights into tumor heterogeneity, vascularity, and molecular composition. From handcrafted texture features and ADC histogram metrics to deep-learning–derived signatures and radiogenomic mappings, imaging biomarkers hold promise for risk stratification and treatment personalization. As the field advances toward standardized, validated, and interpretable models, neuro-imaging will become integral to precision management of brain tumors, enabling clinicians to tailor therapeutic strategies to individual tumor biology and ultimately improve patient outcomes. The articles published in this Research Topic exemplify the strong and sustained interest in emerging technologies applied to the initial assessment, differential diagnosis, and biological characterization of brain tumors — particularly gliomas — and reflect the ongoing efforts of the scientific community to advance neuroimaging tools that enhance prognostication and therapeutic outcomes for patients with brain neoplasms.

